# Diagnostic yield of additional conventional transbronchial lung biopsy following radial endobronchial ultrasound lung biopsy for peripheral pulmonary lesions

**DOI:** 10.1111/1759-7714.13446

**Published:** 2020-04-27

**Authors:** Sojung Park, Hee‐Young Yoon, Yeji Han, Kyung Sook Wang, So Young Park, Yon Ju Ryu, Jin Hwa Lee, Jung Hyun Chang

**Affiliations:** ^1^ Division of Pulmonary and Critical Care Medicine, Department of Internal Medicine, Mokdong Hospital College of Medicine, Ewha Womans University Seoul Republic of Korea; ^2^ Division of Pulmonary and Critical Care Medicine, Department of Internal Medicine Seoul Hospital, College of Medicine, Ewha Womans University Seoul Republic of Korea; ^3^ Bronchoscopy unit Mokdong Hospital, Ewha Womans University Seoul Republic of Korea

**Keywords:** Biopsy, diagnosis, nodule, peripheral, ultrasound

## Abstract

**Background:**

Radial endobronchial ultrasound (R‐EBUS) transbronchial lung biopsy (TBLB) improves the diagnostic yield from peripheral pulmonary lesions (PPLs). However, the small specimens obtained using small forceps through a guide sheath (GS) may impede diagnosis and molecular analysis. Here, we investigated the diagnostic significance of additional conventional TBLB with standard forceps after R‐EBUS‐GS‐guided TBLB.

**Methods:**

We retrospectively reviewed data from 55 patients who underwent conventional TBLB after R‐EBUS‐GS‐guided TBLB for PPL diagnosis. Procedures were performed on single PPLs with no visible lesions on bronchoscopy. In cases with inconclusive pathologic confirmation, final diagnoses were made based on pathologic specimens or clinical observations.

**Results:**

The median size of the target lesions was 28 mm. The appearances on computed tomography images were solid (*n* = 45, 81.8%), part‐solid (*n* = 7, 12.7%), and cavitary nodules (*n* = 3, 5.5%). A computed tomography bronchus sign was present in 35 (63.6%) cases, and a radial probe was positioned within target lesion in 32 (58.2%) cases. R‐EBUS‐GS‐guided TBLB was diagnostic in 30 (54.5%) patients, and subsequent conventional TBLB yielded additional diagnostic information in 8 (14.5%) patients. Probe positioning within target lesions and the outer margin of PPLs more than 1 cm from pleura were significantly associated with enhanced diagnostic yield from the combined procedures. In conventional TBLB, probe positioning within target lesions (75.0% vs. 11.8%, *P* = 0.004) and characteristic of nonsolid nodules (83.3% vs. 15.8%, *P* = 0.006) were significantly associated with additional diagnostic utility.

**Conclusions:**

Conventional TBLB following R‐EBUS‐GS‐guided TBLB could be a useful procedure for diagnosing PPLs, especially for nonsolid nodules.

**Key points:**

**Significant findings of the study:** Additional conventional TBLB with standard forceps after R‐EBUS‐GS‐guided TBLB yielded an additional 14.5% diagnostic utility for peripheral pulmonary lesions. For conventional TBLB, probe positioning within target lesions and nonsolid nodules were significantly associated with additional diagnostic utility.

**What this study adds:** Conventional TBLB with standard forceps after R‐EBUS‐GS‐guided TBLB is an effective and economically accessible diagnostic tool for peripheral pulmonary lesions.

## Introduction

Lung cancer is the leading cause of cancer‐related deaths worldwide. As the survival benefits of chest computed tomography (CT) scans for lung cancer screening have been proven, the detection rate of small peripheral lung nodules has increased.^1^ Diagnostic sampling of these small peripheral pulmonary lesions (PPLs) can usually be achieved via a percutaneous needle biopsy (PCNB). However, the incidence of pneumothorax has been reported to be 17–26.6% in CT‐guided PCNB.^2^, ^3^, ^4^, ^5^, ^6^ Additionally, PCNB cannot be easily performed for PPLs that are surrounded by emphysema or in patients who cannot hold their breath.

Several guided‐bronchoscopy technologies, such as radial endobronchial ultrasound (R‐EBUS), electromagnetic navigation bronchoscopy (ENB), and virtual bronchoscopy (VB), have improved the diagnostic yield of transbronchial lung biopsy (TBLB) for PPL.^7^, ^8^, ^9^, ^10^, ^11^, ^12^, ^13^ However, equipment costs are an important consideration for these methods. ENB and VB both require specialized planning software, and costly hardware. ENB also uses disposable locatable guides, which imposes considerable procedure‐related costs. R‐EBUS only requires an ultrasound and a reusable EBUS probe. In an ordinary hospital setting without expensive navigation equipment, combined R‐EBUS and conventional TBLB could be a better way to improve diagnostic yield than R‐EBUS TBLB alone. Diagnostic yields of R‐EBUS‐guided TBLB have been found to vary widely among previous studies, ranging from 58% to 77%.^7^, ^8^, ^9^, ^14^ A guide‐sheath (GS) improves the diagnostic yield of R‐EBUS‐guided TBLB by keeping the GS within the lesion after the radial probe has been removed.^7^ However, the small forceps that are required to pass through the GS may result in acquiring only a small amount of tissue, which may lead to inadequate specimens for pathologic diagnosis and molecular analysis. The advantage of additional TBLB under fluoroscopy is that the procedure may achieve a higher success rate given the knowledge of the path leading to the lesion through the previous EBUS‐TBLB, and larger tissues can be obtained using relatively large forceps.

We investigated whether subsequent conventional TBLB using larger standard forceps after R‐EBUS‐GS‐guided TBLB could increase the diagnostic yield of PPLs.

## Methods

### Study population

We retrospectively reviewed the medical records of all patients who underwent combined R‐EBUS‐GS‐guided TBLB and subsequent conventional fluoroscopy‐guided TBLB to diagnose PPLs between July 2016 and July 2019. PPLs were defined as lesions surrounded by lung parenchyma and located within the outer one third of the lung. R‐EBUS‐GS‐guided TBLB or CT‐guided PCNB were selected according to the preferences of the attending physicians. However, in cases such as significant cardiovascular disease, hypoxemia, inaccessible bronchial pathway to the target lesion on CT, and PPLs located in apical segments, PCNB was considered first. Patients who had any visible bronchial lesions on conventional bronchoscopy were excluded. To compare the diagnostic yield of the combined procedures with PCNB, we investigated the diagnostic yields and complication rates of patients who underwent PCNB during the same period. The study protocol was approved by the Institutional Review Board of Ewha Womans University Mokdong Hospital (approval number: 201910040).

### Target localization and procedures

CT images of axial, sagittal, and coronal views with a slice thickness of 1.0 mm were obtained to identify small bronchial branching and the bronchial path to a PPL. The window width and window level setting were 1500 Hounsfield units (HU) and −750 HU, respectively. A 4.9 mm bronchoscope (BF‐Q290; Olympus, Tokyo, Japan) was advanced as far as possible into the nearest bronchus to the target lesion after reviewing the paths under the lung window setting of the CT image. Then, a 20‐MHz mechanical radial‐type probe (UM‐S20‐17S, Olympus) connected with EBUS (EU‐M30S, Olympus), which was covered by a GS (SG‐200C, Olympus), were inserted through the bronchoscope working channel of 20 mm. Once the lesion was confirmed by R‐EBUS imaging and X‐ray fluoroscopy, the probe was withdrawn keeping the GS in place. Small biopsy forceps with an outer diameter of 1.5 mm and a cup opening size of 4.0 mm (FB‐233D, Olympus) and a bronchial brush with an outer diameter of 1.4 mm (BC‐204D‐2010, Olympus) were sequentially introduced through the GS to obtain tissue and cytology samples. After finishing R‐EBUS‐GS‐guided TBLB 8–10 times, 2–5 conventional TBLBs using reusable standard biopsy forceps with a 4.0 mm cup opening and a 2.0 mm outer diameter (FB‐19C‐1, Olympus) or disposable forceps with a 5.0 mm cup opening and a 2.0 mm outer diameter (FB‐231D, Olympus) were performed under fluoroscopy. The ultrasound findings were classified into three categories, based on the position of the probe relative to the lesion: within, adjacent to, and invisible.^15^ Bronchoscopies were performed by two bronchoscopists at a time: one fixed and one assistant. All participating bronchoscopists had previously performed more than several hundred conventional bronchoscopies.

As a preliminary examination, we compared the weight of specimens obtained by different forceps using beef. These tests showed that small forceps used in R‐EBUS‐GS obtained 0.60 ± 0.24 mg of beef, FB‐231D forceps obtained 2.51 ± 0.50 mg, and FB‐19C‐1 forceps obtained 0.90 ± 0.16 mg of beef (*P* < 0.001, Table S1).

### Diagnosis

All target lesions were investigated to discriminate malignancy. A diagnosis of malignancy made by the combined procedures was considered true‐positive. In cases of nonmalignant results or inconclusive diagnoses, PCNB was performed to make the definitive diagnosis. In cases where the pathological diagnosis was ambiguous, the final diagnosis was made by surgical biopsy or clinical observation over one year.

### Statistical analysis

Either a Pearson's chi‐square test or the Fisher exact test was used to compare categorical variables. Logistic regression analysis was used for the multivariate analysis of factors that affected the diagnostic yield of the combined procedure. We report the 95% confidence intervals (CIs), and all tests were two‐sided. Differences between groups were considered significant when *P*‐values were < 0.05. All statistical analyses were performed with SPSS software (version 22.0; IBM Corp., Armonk, NY, USA).

## Results

A total of 57 patients underwent the combined procedure of R‐EBUS‐GS‐guided TBLB and subsequent conventional fluoroscopy‐guided TBLB to diagnose PPLs during the study period. After excluding two patients who showed endobronchial lesion on conventional bronchoscopy, 55 patients were ultimately included in the present study. The mean age of the study population was 67.2 years; 29 (52.7%) were male (Table 1). Emphysematous changes in the lung were observed in 10 patients (18.2%); however, these did not surround any target lesions. The median size of target lesions was 28 mm. All target lesions were peripherally located, with a median distance between the outer margin of target lesions and the costal pleural surface of 5 mm; 17 (30.9%) abutted the pleura. Open‐bronchus sign in a target lesion on CT imaging was present in 35 cases (63.6%). The EBUS probe was positioned within the lesion in 32 cases (58.2%), whereas it was adjacent to the lesion on EBUS imaging in 21 cases (38.2%); there were two cases (3.6%) of invisible lesions. Additionally, 297 patients underwent PCNB during the same period. There were no significant differences between the two groups, except for lesion size and imaging features on CT scans.

**Table 1:** Baseline characteristics of patients who underwent endobronchial ultrasound with a guide‐sheath combined with additional conventional transbronchial lung biopsy and percutaneous needle biopsy for peripheral pulmonary lesions

	TBLB	PCNB	
Characteristics	*n* = 55	*n* = 297	*P*‐value
Age, mean (SD)	67 2 (10.9)	68.6 (12.1)	0.417
Male sex	29 (52.7)	173 (58.2)	0.447
Chronic airway disease	13 (23.6)	57 (19.2)	0.448
Emphysema on CT scan	10 (18.2)	75 (25.3)	0.260
Lesion size, median (range)	28 (17–79)	37 (9–137)	0.002
<20 mm	3 (5.5)	32 (10.8)	
20–30 mm	29 (52.7)	77 (25.9)	
>30 mm	23 (41.8)	188 (63.3)	
Lesion location			0.960
Upper lobe	27 (49.1)	152 (51.2)	
Middle or lingular lobe	7 (12.7)	36 (12.1)	
Lower lobe	21 (38.2)	109 (36.7)	
Distance from pleura, mm, median (range)	5 (0–50)	7 (0–70)	0.381
Lesions abutting the pleura	17 (30.9)	104 (35.0)	0.556
Presence of bronchus sign on CT scan	35 (63.6)	170 (57.2)	0.058
Endobronchial ultrasound visualization			
Within	32 (58.2)	NA	
Adjacent	21 (38.2)	NA	
Invisible	2 (3.6)	NA	
Appearance on CT scan			0.033
Solid	45 (81.8)	275 (92.6)	
Part‐solid	7 (12.7)	11 (3.7)	
Pure GGO	0	1 (0.3)	
Cavitary	3 (5.5)	10 (3.4)	

Data are shown as n (%), unless otherwise noted.

CT, computed tomography; GGO, ground‐glass opacity; NA, not applicable; PCNB, percutaneous needle biopsy; SD, standard deviation; TBLB, transbronchial lung biopsy.

**Figure 1:**
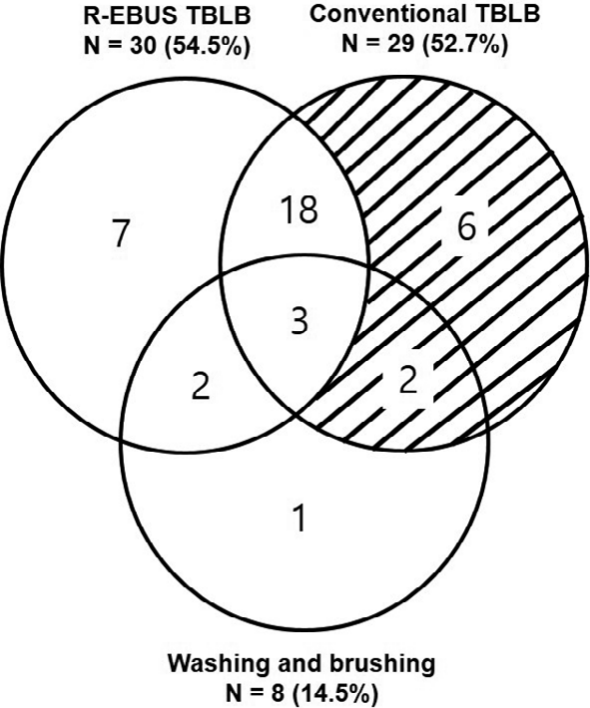
A summary of the diagnostic yield of endobronchial ultrasound with a guide‐sheath, conventional transbronchial lung biopsy, brushing, and their combination for peripheral lung lesions. R‐EBUS, radial endobronchial ultrasound; TBLB, transbronchial lung biopsy.

**Figure 2:**
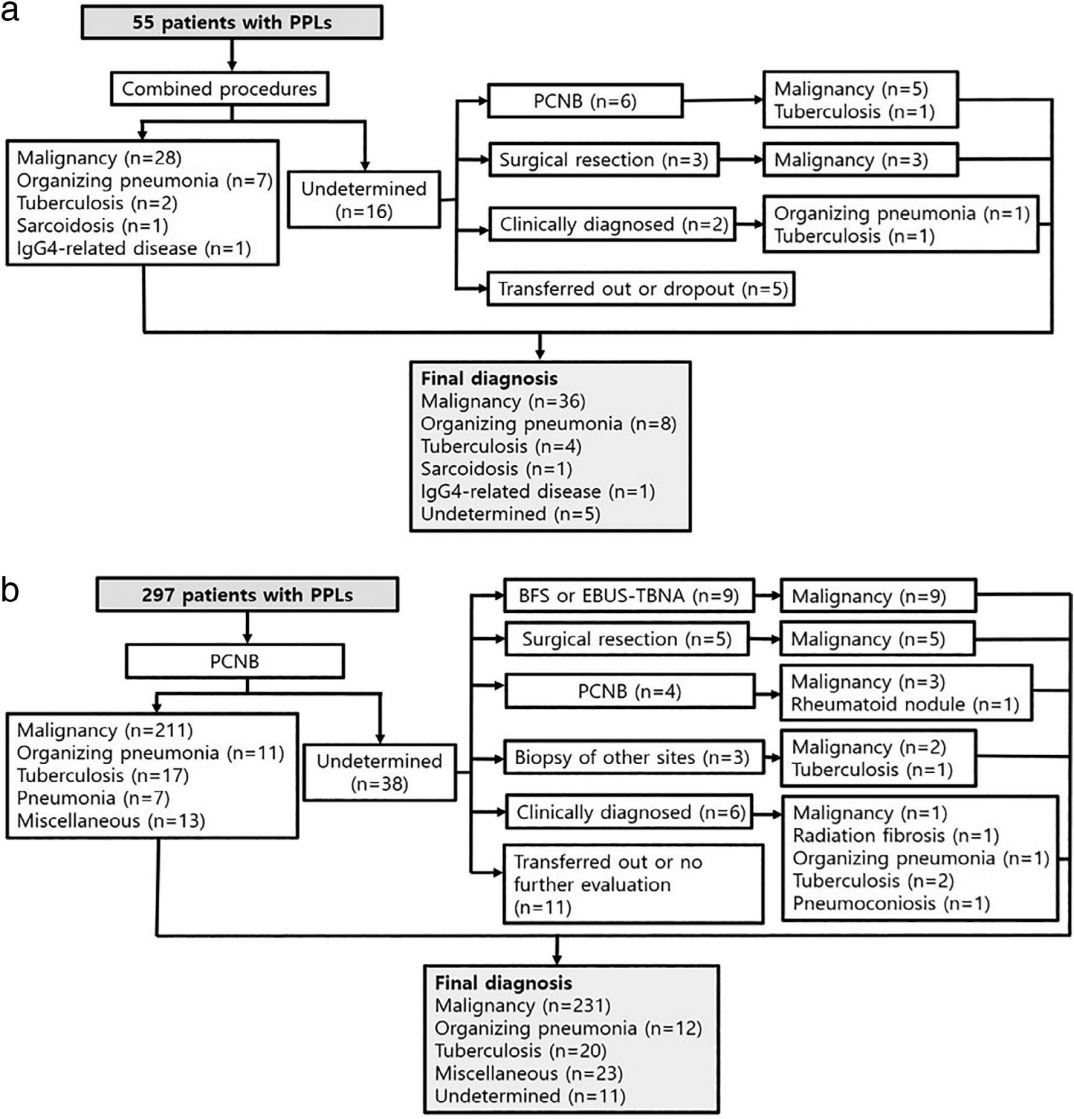
The diagnostic process and final diagnoses of (**a**) 55 patients who underwent endobronchial ultrasound with a guide‐sheath combined with additional conventional transbronchial lung biopsy and (**b**) 297 patients who underwent percutaneous needle biopsy for peripheral pulmonary lesions. PPLs, peripheral pulmonary lesions; PCNB, percutaneous needle biopsy; BFS, bronchofiberscopy; EBUS‐TBNA, endobronchial ultrasound transbronchial needle aspiration.

In total, 39 patients had confirmed diagnoses by the combined procedures. R‐EBUS‐GS‐guided TBLB was initially diagnostic in 30 patients (54.5%), while subsequent conventional TBLB was additionally diagnostic in eight patients (14.5%, Fig. 1). Malignancy was diagnosed in 36 patients (65.5%, Fig. 2a). Among these, 28 (77.8%) were diagnosed by the combined procedures, while patients who showed false negativity were ultimately diagnosed with other modalities: five (13.9%) by PCNB and three (8.3%) by surgical resection. Insufficient amounts of tissue for molecular genetic analysis of epidermal growth factor receptor mutations and anaplastic lymphoma kinase rearrangements, which required additional PCNB, occurred in one case of adenocarcinoma. On the other hand, 259 (87.2%) patients had confirmed diagnoses by PCNB (Fig. 2b). Among the 231 patients who were finally diagnosed with malignancies, 211 (91.3%) were diagnosed with PCNB and nine (9/211, 4.3%) cases failed molecular genetic analysis owing to insufficient amounts of tissue.

A distance of ≥1 cm from the pleura to the outer margin of PPL (94.1% *versus* 60.5%, *P* = 0.012), open bronchus signs on CT images (88.6% vs. 40.0%, *P* < 0.001), and positioning of the radial probe within the target lesion (93.8% vs. 39.1%, *P* < 0.001) were significantly associated with positive results through sequential biopsy procedures (Table 2). Through univariate and multivariate analysis, probe positioning within the target lesion (odds ratio [OR], 32.6; 95% CI, 5.3–199.8, *P* < 0.001) and a distance ≥1 cm from the pleura to the outer margin of PPL (OR, 17.9; 95% CI, 1.6–190.5, *P* = 0.017) were independent factors that influenced the diagnostic yield (Table 3). Although increased distance from the PPL to the pleura was a favorable factor for diagnostic yield, 10 of 17 (58.8%) cases abutting the pleura were diagnostic; there were also seven cases with CT bronchus signs and eight cases with probe positioning within the target lesion.

**Table 2:** Factors associated with the diagnostic yield of endobronchial ultrasound with a guide‐sheath combined with additional conventional transbronchial lung biopsy

	Diagnostic yield	
Characteristics	(*n* = 39/55)	*P*‐value
Location of lesion		0.132
Upper lobe	21/27 (77.8)	
Middle or lingular lobe	6/7 (85.7)	
Lower lobe	12/21 (57.1)	
Lesion size		0.309
≤3 cm	21/32 (65.6)	
>3 cm	18/23 (78.3)	
Distance from pleura		0.012
≤1 cm	23/38 (60.5)	
>1 cm	16/17 (94.1)	
Presence of bronchus sign on CT scan		<0.001
Present	31/35 (88.6)	
Absent	8/20 (40.0)	
Endobronchial ultrasound visualization		<0.001
Within	30/32 (93.8)	
Adjacent to or invisible	9/23 (39.1)	
Appearance on CT scan		0.128
Solid	30/45 (66.7)	
Nonsolid	9/10 (90.0)	

Data are shown as n (%), unless otherwise noted.

CT, computed tomography.

**Table 3:** Logistic regression analysis of factors associated with the diagnostic yield of endobronchial ultrasound with a guide‐sheath combined with additional conventional transbronchial lung biopsy

	Univariate		Multivariate	
Characteristics	Odds ratio (95% CI)	*P*‐value	Odds ratio (95% CI)	*P*‐value
Presence of bronchus sign on CT scan	11.625 (2.946–45.877)	<0.001		
Endobronchial ultrasound visualization	23.333 (4.444–122.510)	<0.001	32.628 (5.328–199.823)	<0.001
Distance from pleura	10.435 (1.249–87.144)	0.030	17.903 (1.628–190.527)	0.017

CI, confidence interval; CT, computed tomography.

Among the 25 patients who could not be diagnosed by R‐EBUS‐GS‐guided TBLB, eight showed additional diagnostic utility from conventional fluoroscopy‐guided TBLB. Nonsolid nodule (83.3% vs.15.8%, *P* = 0.006) and probe positioning within the target lesion (75.0% vs.11.8%, *P* = 0.004) were significantly associated with additional diagnostic utility of conventional TBLB (Table 4).

**Table 4:** Factors associated with the diagnostic utility of additional conventional transbronchial lung biopsy in patients who could not be diagnosed by endobronchial ultrasound with a guide‐sheath‐guided transbronchial lung biopsy

	Diagnostic yield	
Characteristics	(*n* = 8/25)	*P*‐value
Location of lesion		0.345
Upper lobe	5/11 (45.5)	
Middle or lingular lobe	0/2	
Lower lobe	3/12 (25.0)	
Lesion size		1.000
≤3 cm	5/17 (29.4)	
>3 cm	3/8 (37.5)	
Distance from pleura		0.231
≤1 cm	6/22 (27.3)	
>1 cm	2/3 (66.7)	
Presence of bronchus sign on CT scan		0.081
Present	6/11 (54.5)	
Absent	2/14 (14.3)	
Endobronchial ultrasound visualization		0.004
Within	6/8 (75.0)	
Adjacent or invisible	2/17 (11.8)	
Appearance on CT scan		0.006
Solid	3/19 (15.8)	
Nonsolid	5/6 (83.3)	

Data are shown as n (%), unless otherwise noted.

CT, computed tomography.

Fever was the most common complication of TBLB; three patients (5.5%) developed fever during the first 24 hours after the procedure. However, all episodes of fever were resolved spontaneously without any source of infection. There was one episode of pneumothorax, which resolved spontaneously. Meanwhile, there was a significant difference in the incidence of pneumothorax between the groups; there was one case (1.8%) among patients who underwent TBLB and 37 cases (12.5%) among patients who underwent PCNB (*P* < 0.001).

## Discussion

The present study investigated the utility of subsequent conventional TBLB after R‐EBUS‐GS‐guided TBLB for diagnosing PPLs. The combined procedures were diagnostic in 39 (70.9%) patients, including 28 (28/36, 77.8%) patients with malignancy and 11 (11/14, 78.6%) patients with benign diseases; except five patients who were either transferred out or lost to follow‐up. Subsequent conventional TBLB yielded additional diagnostic confirmation in eight (14.5%) patients. Interestingly, three cases (42.9%) of part‐solid ground‐glass nodules (GGNs) were diagnosed only by conventional TBLB.

Previous meta‐analyses have shown that the diagnostic accuracy of R‐EBUS‐GS‐guided TBLB ranged from 68.9% to 74.6%, which is comparable to our results (Table S2).^16^, ^17^, ^18^, ^19^, ^20^ Although it does not involve navigating the bronchoscope to the target lesion, R‐EBUS has been proven to be valuable for confirming the accuracy of biopsy points.^9^ After the lesion is localized and the ultrasound probe is removed, R‐EBUS‐guided TBLB can be performed without losing the position of the nodule by using the GS. In particular, as peripheral lesions display more displacement with breathing compared with central lesions, more significant displacement of biopsy points can occur after the radial probe has been removed in peripherally located lesions. We assumed that GS was more likely to be needed for more peripherally located pulmonary lesions; however, the small forceps that are needed to pass through the GS result in acquiring only a small amount of tissue, which may be inadequate to make a diagnosis and/or for molecular analysis. In the present study, sequential conventional TBLB with larger forceps showed 14.5% additional diagnostic utility. Once the nearest visible bronchial route is established under R‐EBUS guidance, the biopsy forceps in subsequent TBLB follow the route from the previous EBUS procedure to enter the target, making access more accurate.

Previous studies have demonstrated that the optimal diagnostic yield of PPLs can be achieved by taking between three and five biopsy specimens in central lesions, while optimal yields require taking five or more specimens using EBUS‐GS, with the cumulative diagnostic yield reaching to 100% in the tenth specimen.^10^, ^21^, ^22^ It has been estimated that approximately 2–4 fold differences in tissue weights can be extracted from forceps of three different volumes (Table S1). Although the sum of tissue volumes is equal, biopsies using small forceps can increase artifacts in the specimen. Izumo *et al*. reported that rapid on‐site evaluation (ROSE) during EBUS‐GS had a high sensitivity for PPLs; however, its specificity and diagnostic efficacy were lower compared with ROSE during EBUS‐transbronchial needle aspiration.^23^, ^24^ They described that the presence of bronchial ciliated epithelium, bronchial cartilage and abundant inflammatory cells around PPLs made ROSE more difficult for such lesions than for lymph nodes.^23^ The elongated form of specimens from oval shaped biopsy forceps has the advantage that the morphology of the epithelium and submucosa are more intact, and the large forceps used for transbronchial biopsy yield more tissue, including alveolar tissue, than small forceps.^25^, ^26^ The larger specimens obtained with larger forceps may have additional utility for diagnosing PPLs. A larger amount of specimen might be beneficial not only for the pathological diagnosis of lung cancer but also for detailed molecular analyses. Given that a specific method is used for each mutational analysis, a larger amount of tissue is needed to perform multiple mutational analyses, although high‐quality targeted next generation sequencing can be performed using a small amount of tissue, even a liquid biopsy.

Nonsolid nodules were associated with additional diagnostic utility from conventional TBLB. A previous report showed similar results, as GGNs were a significant factor associated with enhanced diagnostic yield in the course of conventional TBLB following EBUS‐TBLB.^27^ Additionally, there have been several studies regarding the diagnostic success for peripheral ground glass opacity dominant lesions by R‐EBUS‐TBLB.^28^, ^29^, ^30^ Interestingly, Ikezawa *et al*. reported that the number of biopsy specimens was an important factor for increasing the diagnostic yield of R‐EBUS‐TBLB in GGNs.^29^ The heterogeneity of EBUS imaging in GGNs may hinder confirming the position of the probe; thus, the large amount of tissue acquired from using larger forceps during conventional TBLB could increase the diagnostic yield.

Various factors, such as lesion size, location, position of the probe, and CT bronchus sign are known to be associated with diagnostic yield of R‐EBUS for PPLs.^10^, ^31^, ^32^ Consistent with previous studies, positioning of the probe within target lesion and location of the lesion were factors associated with an accurate diagnosis in multivariate analysis. We included 38 (69.1%) cases located within 1 cm from the pleura, including 17 (30.9%) cases abutting the pleura. This might have led to the relatively low diagnostic utility of R‐EBUS‐GS‐guided TBLB in our study.

The present study has several limitations. First, we included a small number of patients and this was a retrospective analysis, so there may have been selection bias. Second, we did not compare the diagnostic utility of the combined procedures with R‐EBUS‐GS‐guided TBLB alone; thus, we cannot determine whether the combined procedures were statistically superior to R‐EBUS‐GS‐guided TBLB alone. Third, R‐EBUS‐GS‐guided transbronchial needle aspiration and ROSE, which have the potential to improve the diagnostic yield of EBUS‐TBLB, were not performed in our institute. Finally, we did not measure the time for additional conventional TBLB, which is one of the quality assessment factors of the procedure.

In conclusion, performing conventional TBLB after R‐EBUS‐GS‐guided TBLB for the diagnosis of PPLs could be a useful procedure, especially for nonsolid nodules. The distance between target lesions and the costal pleura, and correct positioning of the EBUS probe within target lesions are key factors that can improve the diagnostic yield of the combined procedures.

## Disclosure

No conflict of interest relevant to this article was reported.

## Supporting information


**Table S1** The weight of beef specimens obtained using different forceps

**Table S2** Meta‐analyses of radial endobronchial ultrasound with a guide sheath guided transbronchial lung biopsy for the diagnosis of peripheral pulmonary lesions
