# Supplementary material for: Diagnostic yield of additional conventional transbronchial lung biopsy following radial endobronchial ultrasound lung biopsy for peripheral pulmonary lesions
Source: Thorac Cancer. 2020 Apr 27;11(6):1639–46. doi: 10.1111/1759-7714.13446 (PMC7263016; doi:10.1111/1759-7714.13446)
Supplement: Supplementary file 1 — Table S1 The weight of beef specimens obtained using different forceps [file TCA-11-1639-s001.docx]

Supplementary Table 1. The weight of beef specimens obtained using different forceps

| Specimen No. | FB-231D | FB-19C-1 | FB-233D | *P*-value |
| --- | --- | --- | --- | --- |
| 1 | 2.89 | 0.96 | 0.57 |  |
| 2 | 2.58 | 1.08 | 0.58 |  |
| 3 | 1.97 | 0.79 | 0.95 |  |
| 4 | 2.76 | 0.85 | 0.79 |  |
| 5 | 2.81 | 1.15 | 0.77 |  |
| 6 | 2.59 | 1.05 | 0.73 |  |
| 7 | 1.93 | 0.9 | 0.68 |  |
| 8 | 3.3 | 0.73 | 0.49 |  |
| 9 | 2.58 | 0.8 | 0.15 |  |
| 10 | 1.69 | 0.66 | 0.33 |  |
| Mean ± SD | 2.51 ± 0.50^†^ | 0.90 ± 0.16^‡^ | 0.60 ± 0.24^†,‡^ | <0.001 |

^†^Statistically significant differences between FB-231D (Olympus, Japan) and FB-233D(Olympus); ^‡^Statistically significant differences between FB-19C-1(Olympus) and FB-233D. SD, standard deviation
