# Supplementary material for: Diagnostic yield of additional conventional transbronchial lung biopsy following radial endobronchial ultrasound lung biopsy for peripheral pulmonary lesions
Source: Thorac Cancer. 2020 Apr 27;11(6):1639–46. doi: 10.1111/1759-7714.13446 (PMC7263016; doi:10.1111/1759-7714.13446)
Supplement: Supplementary file 2 — Table S2 Meta‐analyses of radial endobronchial ultrasound with a guide sheath guided transbronchial lung biopsy for the diagnosis of peripheral pulmonary lesions [file TCA-11-1639-s002.docx]

Supplementary Table 2. Meta-analyses of radial endobronchial ultrasound with a guide-sheath-guided transbronchial lung biopsy for the diagnosis of peripheral pulmonary lesions

| First author | Selected references  within the study | Success cases  per total trials | Diagnostic yield  (ranges) |
| --- | --- | --- | --- |
| Steinfort DP^16^ | 26, 27, 28, 30, 31, 32, 33, 40 | 570/814 | 70.0% (46.0-86.2) |
| Wang Memoli JS^17^ | 10, 14, 15, 20, 30, 32, 35, 40 | 561/800 | 70.1% (58.3-86.2) |
| Ye J^18^ | 6, 9, 10, 11 | 144/193 | 74.6% (64.6-82.5) |
| Ali MS^19^ | 49, 50, 51, 52, 54, 56, 60, 62, 64, 65, 66, 67, 68, 71. 73, 75, 79, 80, 81, 82, 85, 87, 88, 89, 92, 98, 99, 100,101, | 2208/3154 | 70.0% (56.7-85.9) |
| Zhan P^20^ | 17, 19, 22, 23, 24, 25, 28, 32, 34, 35, 36, 38, 40, 41 | 976/1417 | 68.9% (46.0-86.2) |
